# Interleukin-6 increases the expression and activity of insulin-degrading enzyme

**DOI:** 10.1038/srep46750

**Published:** 2017-04-21

**Authors:** Mirian A. Kurauti, José M. Costa-Júnior, Sandra M. Ferreira, Gustavo J. Santos, Carlos H. G. Sponton, Everardo M. Carneiro, Guilherme D. Telles, Mara P. T. Chacon-Mikahil, Cláudia R. Cavaglieri, Luiz F. Rezende, Antonio C. Boschero

**Affiliations:** 1 Obesity and Comorbidities Research Center (OCRC), Institute of Biology, University of Campinas (UNICAMP), Campinas, SP, Brazil; 2 Department of Physiological Sciences, Center of Biological Sciences, Federal University of Santa Catarina (UFSC), Florianopolis, SC, Brazil; 3 Exercise Physiology Laboratory (FISEX), Faculty of Physical Education, University of Campinas (UNICAMP), Campinas, SP, Brazil; 4 Laboratory of Health Sciences, Department of Physiopathology, State University of Montes Claros (UNIMONTES), Montes Claros, MG, Brazil

## Abstract

Impairment of the insulin-degrading enzyme (IDE) is associated with obesity and type 2 diabetes mellitus (T2DM). Here, we used 4-mo-old male C57BL/6 interleukin-6 (IL-6) knockout mice (KO) to investigate the role of this cytokine on IDE expression and activity. IL-6 KO mice displayed lower insulin clearance in the liver and skeletal muscle, compared with wild type (WT), due to reduced IDE expression and activity. We also observed that after 3-h incubation, IL-6, 50 and 100 ng ml^−1^, increased the expression of IDE in HEPG2 and C2C12 cells, respectively. In addition, during acute exercise, the inhibition of IL-6 prevented an increase in insulin clearance and IDE expression and activity, mainly in the skeletal muscle. Finally, IL-6 and IDE concentrations were significantly increased in plasma from humans, after an acute exercise, compared to pre-exercise values. Although the increase in plasma IDE activity was only marginal, a positive correlation between IL-6 and IDE activity, and between IL-6 and IDE protein expression, was observed. Our outcomes indicate a novel function of IL-6 on the insulin metabolism expanding the possibilities for new potential therapeutic strategies, focused on insulin degradation, for the treatment and/or prevention of diseases related to hyperinsulinemia, such as obesity and T2DM.

Interleukin-6 (IL-6) is a pleiotropic cytokine with several functions in different tissues^1^. Initially, IL-6 was described as an important factor of the immune system^2^. However, it has been shown that this cytokine also plays an important role in metabolic regulation^3^, especially on glucose homeostasis^4^.

Insulin is one of the most important hormones in glucose homeostasis, and its action depends on its secretion, the sensitivity of target tissues, and clearance. There are several studies regarding the effects of IL-6 on insulin sensitivity^5^,^6^ and secretion^7^,^8^; however, studies concerning its function on insulin clearance are scarce.

Insulin clearance occurs mainly in the liver, primarily by the action of insulin-degrading enzyme (IDE)^9^. This enzyme, a 110 kDa zinc-metalloprotease, was initially identified as an insulin degrading enzyme^10^. Subsequently, it was found that IDE also degrades other amyloidogenic peptides, such as amyloid β^11^. Impairment of IDE are closely related to the development of diseases, such as type 2 diabetes mellitus (T2DM)^12^,^13^ and Alzheimer’s disease (AD)^14^. Also, it is proposed that IDE malfunction could be the link between these two pathologies^15^.

Selective modulators of IDE activity could work as potential drugs for treatment of T2DM and AD^16^. While IDE activators have been proposed as AD therapies^17^, it is uncertain if activation of this enzyme would be the better therapeutic approach for T2DM. To the contrary, treatment with IDE inhibitors seems to potentiate insulin signaling^18^ and a fast and short pre-meal IDE inhibition could be useful for T2DM therapy^19^. However, despite increasing insulin signaling, acute IDE inhibition impaired glucose tolerance in mice, casting a shadow on the usefulness of the IDE inhibition for the treatment of T2DM^20^. Interestingly, IDE knockout (KO) mice display chronic hyperinsulinemia^21^ that, over time, induces a reduction of insulin receptor expression, leading to insulin resistance^22^. Also, downregulation of IDE, associated with hyperinsulinemia, is observed in obese and diabetic patients^23^,^24^, and rodents^13^,^25^. Therefore, we believe that finding molecules which are able to increase IDE function could be important for the development of new therapeutic strategies against diseases related to hyperinsulinemia, such as obesity and T2DM.

Some interventions^25^,^26^,^27^ and molecules^28^,^29^ can modulate the expression and activity of IDE. In this way, physical exercise increases IDE expression in the liver and skeletal muscle of rodents^30^, and this could explain the augmentation of insulin clearance observed in these rodents and humans^31^. It is also known that the ciliary neurotrophic factor (CNTF), a member of the IL-6 family of cytokines, can also modulate insulin clearance and IDE expression in the liver of Swiss mice and in HEPG2 cells^32^. Thus, we hypothesized that IL-6 likewise could have an effect on insulin degradation.

Here, we demonstrate that IL-6 deficient mice displayed reduced insulin clearance, probably due to lower IDE action in the liver and skeletal muscle. In addition, IL-6 incubation increased the expression of IDE in HEPG2 and C2C12 cells. We also found that, during acute endurance exercise, IL-6 mediated the increase of IDE expression and activity, mainly in the skeletal muscle, increasing insulin clearance, a phenomenon that may also occur in humans.

## Results

### IL-6 KO mice displayed altered metabolic parameters

KO mice showed a significant reduction of IL-6 content in plasma, liver, and skeletal muscle, confirming the deficiency of this cytokine in these mice (Suppl Fig. 1). KO mice also displayed a reduction in the body and skeletal muscle weight, despite an augmented adiposity, compared with wild type (WT) mice. In addition, a decreased insulinemia and increased glycemia was observed in the KO group (Table 1).

### IL-6 KO mice had impaired glucose, but not insulin, tolerance

**Figure 1:**
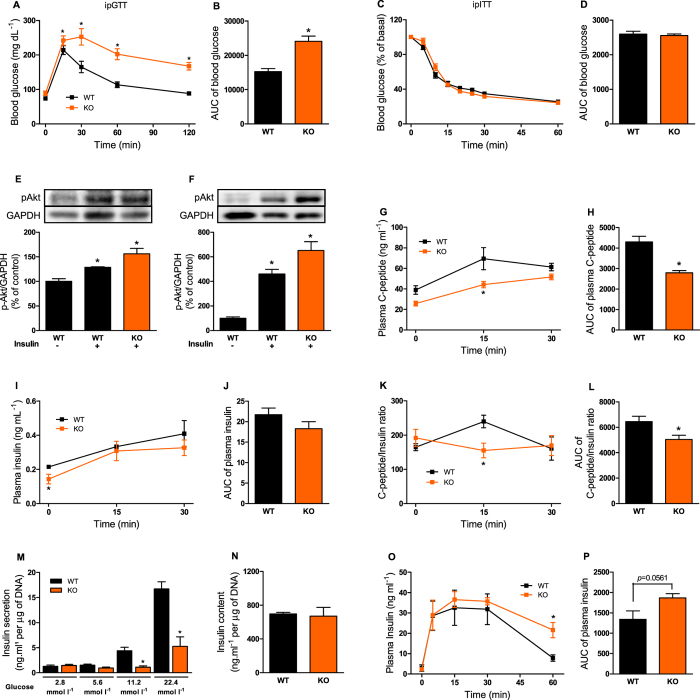
Glucose and insulin metabolism in the IL-6 KO mice. Blood glucose (**A**) and AUC of blood glucose (**B**) before (0 min) and 15, 30, 60 and 120 min after administration of 1 g kg^−1^ glucose, n = 6. Blood glucose (**C**) and AUC of blood glucose (**D**) before (0 min) and 5, 10, 15, 20, 25, 30 and 60 min after administration of 0.75 U kg^−1^ insulin, n = 6. Immunoblotting of AKT phosphorylated at serine 473 with (+) or without (−) administration of 10 U insulin per mouse, in the liver (**E**) and gastrocnemius muscle (**F**), n = 4. Plasma C-peptide (**G**), insulin (**I**) and C-peptide/insulin ratio (**K**), during ipGTT, and its respective AUCs (**H,J** and **L**), n = 4. Insulin secretion in isolated pancreatic islets stimulated with 2.8, 5.8, 11.2 and 22.4 mmol l^−1^ glucose (**M**). Total insulin content of islets (**N**), n = 7. Plasma insulin concentration (**O**) and AUC of plasma insulin (**P**), during ipITT, n = 6. WT, wild type mice; and KO, IL-6 knockout mice. Data are presented as the mean ± S.E.M. *p ≤ 0.05 *vs* WT.

IL-6 plays an important role on glucose metabolism^4^,^33^. Here, we observed an impaired glucose tolerance in KO mice (Fig. 1A and B), without a change in insulin tolerance (Fig. 1C and D), compared with WT mice. In addition, the Akt phosphorylation in the liver and skeletal muscle (Fig. 1E and F) was similar between KO and WT groups.

### IL-6 KO mice displayed decrease of insulin secretion and clearance

During the ipGTT, KO mice displayed decreased plasma C-peptide concentration (Fig. 1G and H), indicating impairment of insulin secretion. This observation was confirmed by a significant reduction in glucose-stimulated insulin secretion in isolated pancreatic islets from KO mice (Fig. 1M). Interestingly, the insulinemia was not reduced at the same extent than plasma C-peptide, during the ipGTT (Fig. 1I and J). Thus, the C-peptide/insulin ratio was reduced in the KO mice (Fig. 1K and L), indicating that insulin clearance was also decreased. A higher concentration of insulin was registered at 60 min of the ipITT, in the KO mice (Fig. 1O), indicating a reduction of insulin clearance in these mice, although no significantly difference was observed in the area under curve (AUC) of plasma insulin between groups (Fig. 1P).

### IL-6 KO mice had reduced IDE expression and activity in the liver and skeletal muscle

**Figure 2:**
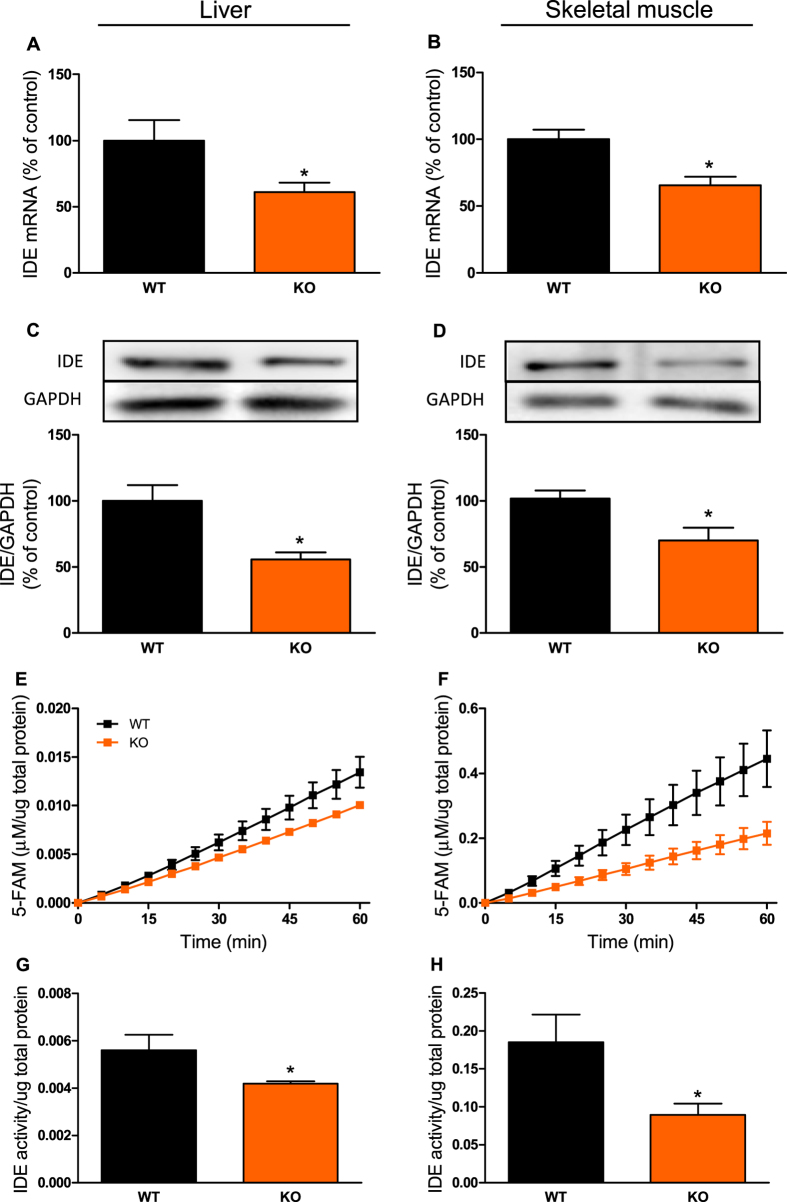
Expression and activity of IDE in the liver and skeletal muscle from IL-6 KO mice. mRNA expression of IDE in the liver (**A**) and gastrocnemius muscle (**B**), n = 6. Protein expression of IDE in the liver (**C**) and gastrocnemius muscle (**D**) and its representative immunoblottings images, n = 5. Kinetic of IDE activity assay in liver (**E**) and in gastrocnemius (**F**) of mice, n = 5–6. Fluorescent intensity at Ex/Em = 490/520 nm was continuously recorded, every 5 min, during 60 min. 5-FAM concentration was calculated using a standard curve and normalized per μg of total protein. IDE activity was calculated as described^27^ and normalized per μg of total protein in the liver (**G**) and gastrocnemius (**H**) of mice, n = 5–6. WT, wild type mice; and KO, IL-6 knockout mice. Data are presented as the mean ± S.E.M. *p ≤ 0.05 *vs* WT.

IDE is the main enzyme responsible for insulin degradation^9^. Corroborating the reduced insulin clearance in the KO mice, IDE gene expression (Fig. 2A and B), protein content (Fig. 2C and D), and activity (Fig. 2E–H) were decreased in the liver and skeletal muscle, compared with WT mice.

### IL-6 increased IDE expression in HEPG2 and C2C12 cells

**Figure 3:**
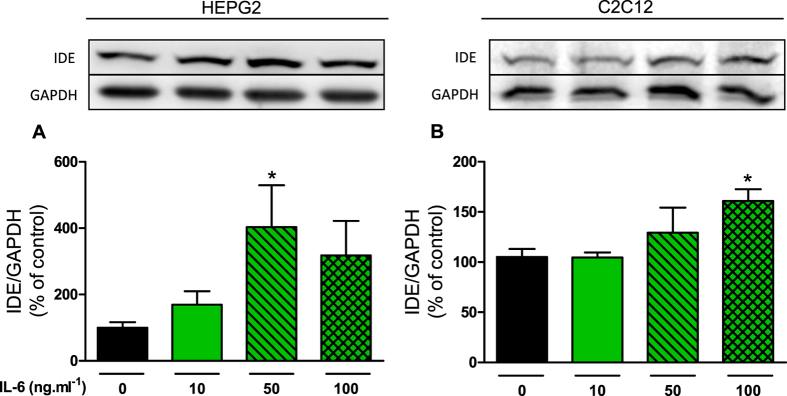
Effects of IL-6 incubation on IDE protein expression in HEPG2 and C2C12 cells. Protein expression of IDE in HEPG2 (**A**) and C2C12 (**B**) cells and its representative immunoblottings images. The cells were incubated at 0, 10, 50 and 100 ng ml^−1^ IL-6, during 3 h. Data are presented as the mean ± S.E.M. n = 3–4. *p ≤ 0.05 *vs* 0 ng ml^−1^ IL-6.

In the following set of experiments, HEPG2 and C2C12 cells were incubated, for 3-h, at different concentrations of IL-6. The IDE protein content was significantly increased in HEPG2 and C2C12 cells, at 50 and 100 ng ml^−1^ IL-6, respectively (Fig. 3A and B).

### IL-6 inhibition impaired the increase on insulin clearance in mice, after an acute endurance exercise

**Figure 4:**
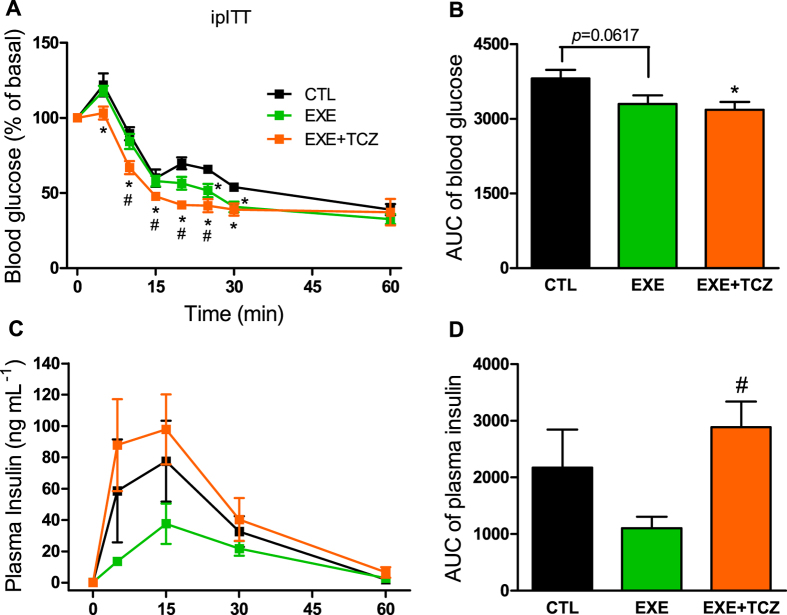
Effect of IL-6 inhibition on insulin tolerance and clearance after acute exercise in mice. Blood glucose (**A**) and AUC of blood glucose (**B**) before and 5, 10, 15, 20, 25, 30 and 60 min after administration of 0.75 U kg^−1^ insulin. Plasma insulin (**C**) and AUC of plasma insulin (**D**) at 0, 5, 15, 30 and 60 min during ipITT. CTL, control mice; EXE, exercised mice; and EXE + TCZ, exercised mice treated with 2 mg kg^−1^ Tocilizumab 1-h before the exercise protocol. Data are presented as the mean ± S.E.M. n = 6. *p ≤ 0.05 *vs* CTL and ^#^p ≤ 0.05 *vs* EXE.

Since a substantial amount of IL-6 is released in the plasma during skeletal muscle contractions (Supple Fig. 2), we investigated the role of this cytokine on insulin clearance, after acute exercise. During ipITT, we observed that inhibition of IL-6 increased insulin tolerance in EXE + TCZ (exercised mice treated with tocilizumab, a pharmacological IL-6 receptor neutralizing antibody), and this increase was bigger than in the exercised (EXE) mice, as judged by the AUC of blood glucose (Fig. 4A and B). Interestingly, the higher insulin tolerance was accompanied by higher levels of plasma insulin during the ipITT, indicating a lower insulin clearance in the EXE + TCZ mice, compared with EXE mice (Fig. 4C and D).

### IL-6 inhibition impaired IDE expression and activity in the skeletal muscle of mice, after an acute endurance exercise

**Figure 5:**
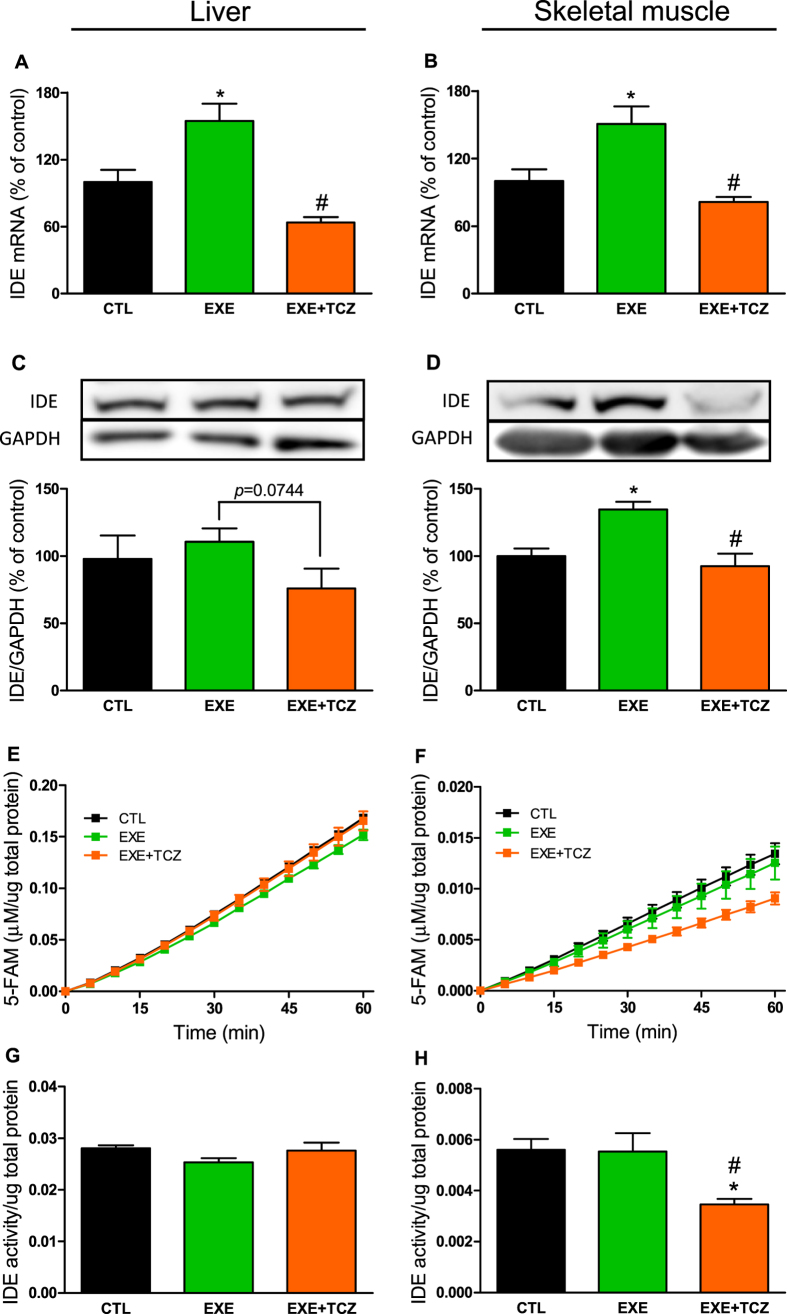
Effects of IL-6 inhibition on IDE expression and activity in the liver and skeletal muscle of mice after acute exercise. mRNA expression of IDE in the liver (**A**) and gastrocnemius muscle (**B**), n = 5–6. Protein expression of IDE in the liver (**C**) and gastrocnemius muscle (**D**) and its representative immunoblottings images, n = 5–6. Kinetic of IDE activity assay in liver (**E**) and gastrocnemius (**F**), n = 5. Fluorescent intensity at Ex/Em = 490/520 nm was continuously recorded, every 5 min, during 60 min. 5-FAM concentration was calculated using a standard curve and normalized per μg of total protein. IDE activity was calculated as described^27^ and normalized per μg of total protein in the liver (**G**) and gastrocnemius (**H**) of mice, n = 5. CTL, control mice; EXE, exercised mice; and EXE + TCZ, exercised mice treated with 2 mg kg^−1^ Tocilizumab 1-h before the acute exercise protocol. Data are presented as the mean ± S.E.M. *p ≤ 0.05 *vs* CTL and ^#^p ≤ 0.05 *vs* EXE.

We also evaluated the IDE expression and activity in exercised mice, treated with an IL-6 inhibitor. Corroborating the insulin clearance data, a single bout of exercise increased IDE gene expression in the liver and skeletal muscle (Fig. 5A and B), but IDE protein expression increased only in the skeletal muscle of EXE mice, compared with CTL mice (Fig. 5C and D). No alteration was found in IDE activity, after acute exercise (Fig. 5E–H). Interestingly, IL-6 inhibition prevents an increase in the expression of the IDE gene in liver and skeletal muscle (Fig. 5A and B), and IDE protein expression only in the skeletal muscle (Fig. 5C and D). We also observed reduced IDE activity in the skeletal muscle of EXE + TCZ mice, compared with EXE mice (Fig. 5F and H).

### IL-6 was positively correlated with IDE protein expression and activity in the plasma of humans subjects, after an acute endurance exercise

**Figure 6:**
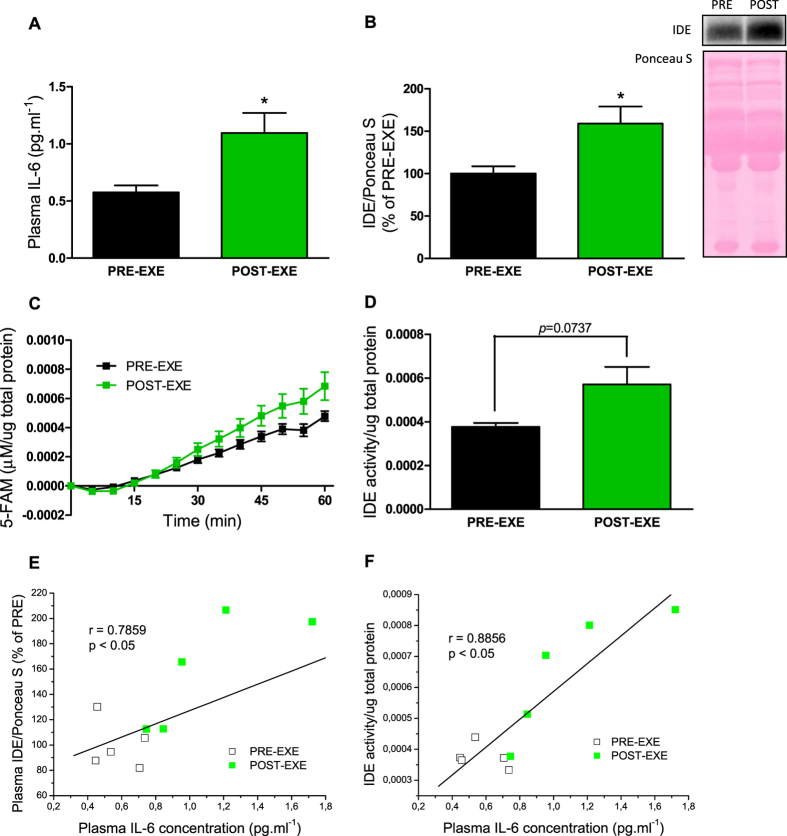
IL-6 concentration and IDE protein expression and activity in the plasma of human subjects, before and after acute endurance exercise. Plasma IL-6 concentration (**A**), n = 5. IDE protein expression and its representative immunoblotting image (**B**), n = 5. Kinetic of IDE activity assay in the plasma (**C**), n = 5. Fluorescent intensity at Ex/Em = 490/520 nm was continuously recorded, every 5 min, during 60 min. 5-FAM concentration was calculated using a standard curve and normalized per μg of total protein. Total IDE activity (**D**) was calculated as described^27^ and normalized per μg of total protein, n = 5. Data represent the mean ± S.E.M. *p ≤ 0.05 *vs* PRE-EXE. Correlation between plasma IL-6 concentration and IDE protein expression (**E**) or IDE activity (**F**), n = 10. The Pearson product moment correlation coefficient (r) was determined using the software GraphPad Prism 5. PRE-EXE, plasma samples of human subjects before acute endurance exercise; and POST-EXE, plasma samples of human subjects 3-h after acute exercise (30-min cycling at 70% of VO_2peak_).

Finally, we measured IL-6 concentration, IDE protein expression and activity in the plasma of human subjects, before and after acute exercise. Plasma IL-6 and IDE concentrations were significantly increased post-exercise, compared with pre-exercise group (Fig. 6A and B), and a positive correlation between these two plasmatic parameters was confirmed (Fig. 6E). Although the increase of IDE activity, in the post-exercise group, was not statistically significant compared with pre-exercise (Fig. 6C and D), this plasmatic parameter was also positively correlated with IL-6 concentration (Fig. 6F).

## Discussion

Impaired IDE expression and/or activity are closely related to diseases associated with hyperinsulinemia, such as obesity and T2DM^12^. Therefore, increasing the function of IDE could be a strategy for the treatment of these pathologies. Here, we found that exposure to IL-6 increased IDE protein expression in HEPG2 and C2C12 cells. We also observed that IL-6 KO mice displayed reduced insulin clearance, probably due to lower IDE function in the liver and skeletal muscle. Finally, we demonstrated that during acute exercise, IL-6 release seems to be necessary to increase IDE expression and activity, mainly in the skeletal muscle of mice, an effect that likely occurs also in humans.

IL-6 is an interesting cytokine with a dual effect on glucose homeostasis. Initially, it was thought that this cytokine has deleterious effects on glucose homeostasis by impairing the insulin signaling, leading to insulin resistance^6^. Later, it was found that the loss of IL-6, not only failed to protect against these deleterious effects, but also induced glucose intolerance leading to development of mature-onset obesity^34^. These data show that IL-6 is important for the maintenance of normal carbohydrate and lipid metabolism. Indeed, here, 4-mo-old IL-6 KO mice developed glucose intolerance (Fig. 1A and B) and increased fat pad weight (Table 1), although impairment in insulin signaling and overweight were not observed. These last alterations were registered in older (9-mo-old) IL-6 deficient mice^34^. Although young IL-6 KO mice do not have impaired insulin sensitivity (Fig. 1C and D), they display reduced glucose-stimulated insulin secretion (Fig. 1M), which could explain the glucose intolerance during ipGTT. These data suggest that the impairment of insulin secretion and/or clearance precede the onset of insulin resistance in IL-6 KO mice.

The role of IL-6 on insulin secretion has been extensively studied, and there is substantial evidence that this cytokine increases insulin secretion^7^,^8^, and this increase seems to depend on the activation of the phospholipase C (PLC) - inositol triphosphate (IP_3_) dependent pathway^8^. However, further investigation demonstrated that IL-6 enhances insulin release also by increasing the glucagon-like peptide 1 (GLP-1) secretion from L cells and pancreatic α cells^7^.

While several studies have investigated the function of IL-6 on insulin secretion, its participation on insulin clearance was neglected. Here, we provide evidence that IL-6 must be essential to enhance insulin clearance probably by increasing IDE expression and activity in the liver and skeletal muscle. These are opposite effects from those reported for CNTF, a member of the IL-6 family of cytokines^32^. Although these two cytokines act through activation of glycoprotein 130 (gp 130), there are some peculiarities in their receptors^35^ that could explain these different effects on insulin clearance and IDE expression.

It is known that increase of adiposity is associated with decrease of IDE expression and insulin clearance, as observed in obesity^25^,^36^. Here, we show that IL-6 KO mice had an increased fat pad weight which could explain the reduced IDE expression and activity in these mice. However, to prove that this IDE modulation is an effect of IL-6 loss, we treated this KO mice with 200 ng IL-6 during 3 days before the experiments. Interestingly, IL-6 treatment restored IDE gene expression of KO mice, without any alteration in the fat pad weight (see Supple Fig. 3). These data, associated with the results from *in vitro* experiments, confirm the important role of IL-6 on IDE modulation.

During physical exercise, muscle contractions release and increase the concentration of IL-6 in the bloodstream, and this myokine^37^ modulates glucose homeostasis. Previous data suggest that the primary function of IL-6 during exercise is to maintain glucose turnover^4^. At the same time that IL-6 favors insulin to stimulate glucose uptake by the skeletal muscle^38^, it increases glucose production in the liver^39^ to sustain the high demand for glucose by the former tissue. Although controversial^40^, it has been reported that, during exercise, this myokine could improve insulin sensitivity^41^. However IL-6 has been found to increase insulin secretion after exercise^7^ whereas a reduction in plasma insulin was observed^42^. Here we proposed that IL-6 could also increase insulin clearance and IDE expression, induced by exercise, as reported^30^. Interestingly, we confirmed our hypothesis showing that inhibition of IL-6 signaling during acute endurance exercise blocks the augmentation of insulin clearance and IDE expression, mainly in the skeletal muscle. This phenomenon seems to be important to avoid a hazardous decrease of glycemia during exercise, making it more effective and safe.

Physical exercise is one of the first clinical approaches to prevent and treat metabolic diseases, such as obesity and T2DM. However, adherence to physical activity by obese and diabetic patients is very low^43^. Thus, finding alternatives for physical exercise would be useful for the prevention and treatment of these diseases. In this scenario, IL-6 has been suggested as a promising candidate^44^. Here, we found that, as in mice, IL-6 seemed to have the same effects on insulin clearance in humans after acute endurance exercise, since the concentration of this myokine positively correlated with IDE expression in the plasma of these subjects. Although considering IL-6 as a promising alternative to physical exercise, treatment with this cytokine may be not recommended due to its dual effects on glucose metabolism. Thus, find the mechanism activated by IL-6 during exercise, could help us to develope a safer exercise mimetic.

IL-6 may act on its target cells by canonical and non-canonical pathways. The canonical pathway is well-established and comprises the activation of the signal transducer and activator of transcription 3 (STAT3), which, in the nucleus, enhances the transcription of several genes^45^,^46^. The non-canonical pathway refers to the activation of the 5′-adenosine monophosphate-activated protein kinase (AMPK). Even though the non-canonical pathway remains poorly understood, it was reported that IL-6 directly activates AMPK through increasing cyclic adenosine monophosphate (cAMP) and AMP/ATP ratio in skeletal muscle of rats^47^. Since IL-6 KO mice show reduced activation of AMPK^48^, we speculate that this non-canonical pathway could be the mechanism whereby IL-6 increases IDE expression. Moreover, we previously demonstrated that the treatment with 5-aminoimidazole-4-carboxamide ribonucleotide (AICAR) increased IDE expression in C2C12 cells^30^, corroborating our hypothesis. However, this data does not exclude the possible role of STAT3 on IDE expression. Therefore, further investigations are necessary to clarify this matter.

In summary, here we described a novel function of IL-6 on glucose homeostasis, which consists of increase insulin clearance, probably due to augmentation of IDE expression and activity in the liver and skeletal muscle. In addition, we demonstrated that, after acute endurance exercise, IL-6 mediated the increase of IDE, mainly in the skeletal muscle of mice, an effect that seems to occur also in humans. Our findings expand the possibilities for new potential therapeutic strategies, focused on insulin degradation, that could be used in the treatment and/or prevention of diseases related to hyperinsulinemia, such as obesity and T2DM.

## Methods

### Animals

The 4-mo-old males C57BL/6 wild type (WT) and IL-6 knockout (KO) mice were maintained on a 12-h light/12-h dark cycle with controlled temperature and humidity during the entire experiment. All mice were allowed to feed (standard chow diet) and drink tap water *ad libitum*. After the experiments using WT and KO mice, other males C57BL/6 WT mice were distributed into three different groups: control (CTL), exercised (EXE) and exercised mice treated with 2 mg kg^−1^ tocilizumab (a pharmacological IL-6 receptor neutralizing antibody) 1-h before the exercise protocol (EXE + TCZ).

### Maximum oxygen consumption (VO_2_ max) and acute endurance exercise protocol in mice

Before the acute exercise protocol, we measured the VO_2_ max in all mice from the CTL, EXE and EXE + TCZ groups, as described before^30^. After 10 days, mice from EXE and EXE + TCZ groups were submitted to a single bout of exercise on a treadmill inclined at 25° for 3-h at 60–70% of VO_2_ max. All measurements made in these mice were performed immediately after the acute exercise.

### Intraperitoneal glucose and insulin tolerance tests (ipGTT and ipITT)

The ipGTT and ipITT was performed as previously described^27^. The blood glucose concentration was measured by tail bleed using glucose strips on Accu-Chek Performa II glucometer (Roche, Sao Paulo, Brazil). During the ipGTT, blood samples were collected from the tail before (0 min) and 15 and 30 min after glucose administration. The plasma samples was obtained by centrifugation (1100 g for 15 min at 4 °C) and was stored at −80 °C for posterior insulin and C-peptide quantification.

### *In vivo* insulin clearance test

During the ipITT, blood samples were collected before (0 min) and 15, 30 and 60 min after insulin administration to determine the plasma insulin concentrations^25^.

### Plasma C-peptide and insulin concentration

Plasma C-peptide was measured by Rat/Mouse C-Peptide 2 ELISA Kit (Cat. EZRMCP2-21K, EMD Millipore), following the manufacturer’s instructions, and plasma insulin concentration was determined by radioimmunoassay (RIA), as described^49^.

### Tissue samples

The mice were killed in a CO_2_-saturated chamber followed by beheading. Liver and gastrocnemius skeletal muscle samples from the mice were collected, snap-frozen in liquid nitrogen, and stored at −80 °C for subsequent experiments (RT-PCR Real time, Western blot and IDE activity). The pancreatic islets isolation was performed as previously described^50^.

### Glucose-stimulated insulin secretion in pancreatic islets

The islets were pre-incubated for 1-h in Krebs-Henseleit buffer solution (KHBS) containing 0.5 g l^−1^ bovine serum albumin (BSA) and 5.6 mmol l^−1^ glucose (95% O_2_, 5% CO_2_, pH 7.4, 37 °C). Subsequently, we incubate five islets per well for an additional hour in the KHBS containing 2.8, 5.6, 11.2 or 22.4 mmol l^−1^ glucose. The supernatant was collected to evaluate the insulin secretion, and the islets were homogenized in an alcohol/acid solution to measure the total insulin content by RIA.

### Real time RT- PCR

Tissues samples of liver and skeletal muscle was homogenized in 1 ml TRIzol^®^ Reagent (Cat. 15596026, Invitrogen™, Thermo Fisher Scientific Inc, Waltham, MA, USA), and the total mRNA was extracted following the manufacturer’s instructions. To prepare the cDNA, we used 1 μg of total mRNA and High-Capacity cDNA Reverse Transcription Kit (Cat. 4368814, Applied Biosystems™, Thermo Fisher Scientific Inc, Waltham, MA, USA). Real time PCR was performed on 7500 Fast Real-time PCR System (Applied Biosystems™) using Fast SYBR^®^ Green Master Mix (Cat. 4385612, Applied Biosystems™). Mice IL-6 and IDE gene expression was measured and GAPDH was used as housekeeping gene (IL-6 forward 5′-CACGGCCTTCCCTACTTCAC-3′ and reverse 5′-GGTCTGTTGGGAGTGGTATC-3′; IDE forward 5′-CTGTGCCCCTTGTTTGATGC-3′ and reverse 5′-GTTCCCCGTAGCCTTTTCCA-3′; GAPDH forward 5′-CCTGCACCACCAACTGCTTA-3′ and reverse 5′-GCCCCACGGCCATCACGCCA-3′). Real-time PCR data were analyzed using the 7500 Software version 2.0.5 (Applied Biosystems™).

### Western Blot

For protein extraction, tissue samples of liver and skeletal muscle, were homogenized in a lysis buffer containing 10 mmol l^−1^ EDTA, 100 mmol l^−1^ Tris base, 100 mmol l^−1^ sodium pyrophosphate, 100 mmol l^−1^ sodium fluoride, 10 mmol l^−1^ sodium orthovanadate, 2 mmol l^−1^ Phenylmethylsulfonyl fluoride, 1% Triton X-100 and 1 μg ml^−1^ aprotinin. Protein concentration of the samples was determined using Bradford reagent (Cat. 500-0006 N, BioAgency Biotecnologia, São Paulo, Brazil). After, 30 μg protein samples were homogenized and boiled (5 min at 100 °C) in a Laemmli buffer, applied on 10% SDS-PAGE (sodium dodecyl sulfate polyacrylamide gel electrophoresis), and transferred to nitrocellulose membranes. These membranes were blocked in a Tris-buffered saline (10 mmol l^−1^ Tris base, 150 mmol l^−1^ NaCl and 0.25% (vol./vol.) of Tween 20) containing 5% (wt./vol.) BSA for 1-h at room temperature. After, the membranes were incubated overnight at 4 °C with primary antibodies (anti-phospho-Akt^ser473^, Santa Cruz Biotechnology cat. sc-7985; anti-IDE, Abcam cat. ab32216; anti-GAPDH, Sigma cat. G9545). Bands detection was performed by chemiluminescence (SuperSignal West Fento, Pierce Biotechnology Inc., Rockford, IL, USA) after incubation with an appropriated horseradish peroxidase-conjugated secondary antibody, and the bands were visualized using the Amersham Imager 600 (GE Healthcare Life Sciences, Pittsburgh, PA, USA). Band intensities were analyzed using ImageJ software (National Institutes of Health, Maryland, USA).

### HEPG2 and C2C12 cells culture

HEPG2 (a human liver carcinoma cell line) and C2C12 (a mouse myoblast cell line) cells were culture as described before^30^. For the differentiation of C2C12 cells, we used DMEM high glucose containing 2% (vol./vol.) horse serum, and we culture it in this medium for 5 days. HEPG2 and differentiated C2C12 cells were incubated at 0, 10, 50 or 100 ng ml^−1^ IL-6 (Cat. PMC0061, Gibco™, Thermo Fisher Scientific Inc, Waltham, MA, USA) for 3-h. The cells were collected in trypsin/EDTA solution, washed with phosphate-buffered saline (PBS), and then homogenized in a urea anti-protease/anti-phosphatase buffer, for subsequent analysis of IDE protein expression.

### Plasma samples of human subjects

Plasma samples of human subjects were obtained from a previous study, and for all detailed information about this subjects (untrained healthy male) and experimental procedures, see ref. 51. The exercise protocol comprise a standardized warm-up period following by 30-min of cycling, at 70% of VO_2peak_. Blood samples were collected before (PRE-EXE) and 3-h after (POST-EXE) the exercise session for posterior analysis of IL-6 concentration, IDE protein expression and activity.

### IDE activity assay

IDE activity, from tissues and plasma samples, was measured by SensoLyte^®^ 520 IDE Activity Assay Kit (Cat. AS-72231, AnaSpec, Fremont, CA, USA), following the manufacturer’s recommendations. The total IDE activity was calculated as previously described^27^, using this equation:


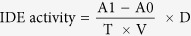


A1 is the concentration of 5-FAM at 60 min and A0 at 0 min; T is the total time of the assay (60 min); V is the volume of samples, and D is the dilution. The 5-carboxyfluorescein (5-FAM, the product of the enzyme reaction) concentration and the total IDE activity were normalized *per* μg of total protein, which was determined using Bradford reagent.

### Quantification of plasma IL-6 concentration

Plasma IL-6 concentrations were measured using Mouse IL-6 ELISA Kit (Cat. EZMIL6, Merck Millipore, Darmstadt, Germany) and Human IL-6 Quantikine^®^ ELISA Kit (Cat. D6050, R&D Systems, Minneapolis, MN, USA), according to the manufacturer’s instructions.

### Statistics

For the statistical analysis of two groups we used the Student’s *t*-test and for three or more groups, we performed the one-way ANOVA using the unpaired Tukey’s *post-hoc* test. The data are presented as the mean ± standard error mean (SEM) and all data were considered significantly different if p ≤ 0.05. The Pearson product moment correlation coefficient (r) was determined to examine the relationship between IL-6 concentration and IDE protein expression or activity in the plasma of human subjects.

### Study approval

All experimental procedures made in mice and humans subjects were approved by the local ethics committee (approval numbers 3087-1 and 848.145). The animal experiments were conducted following to the last revision of the National Institutes of Health (NIH) guide for the care and use of laboratory animals. In the case of humans, the experimental procedures were explained to all subjects, who provided written informed consent before the study that was conducted according to the latest revision of the Declaration of Helsinki^51^.

## Additional Information

**How to cite this article**: Kurauti, M. A. *et al*. Interleukin-6 increases the expression and activity of insulin-degrading enzyme. *Sci. Rep.* **7**, 46750; doi: 10.1038/srep46750 (2017).

**Publisher's note:** Springer Nature remains neutral with regard to jurisdictional claims in published maps and institutional affiliations.

## Supplementary Material

Supplementary Information

## Figures and Tables

**Table 1:** Metabolic parameters of the IL-6 KO mice.

Parameters	WT	KO
Body weight (g)	28.64 ± 0.49	24.79 ± 0.31*
Skeletal muscle pad (% of body weight)	1.139 ± 0.026	1.057 ± 0.016*
Fat pad (% of body weight)	1.046 ± 0.13	1.741 ± 0.11*
Fasting blood glucose (mg dl^−1^)	73.86 ± 4.10	92.29 ± 3.69*
Fasting plasma insulin (ng ml^−1^)	0.265 ± 0.051	0.120 ± 0.009*

*p ≤ 0.05 *vs* WT.
