# Supplementary material for: Interleukin-6 increases the expression and activity of insulin-degrading enzyme
Source: Sci Rep. 2017 Apr 21;7:46750. doi: 10.1038/srep46750 (PMC5399448; doi:10.1038/srep46750)
Supplement: Supplementary Information [file srep46750-s1.pdf]

Supplementary Figures

Interleukin-6 increases the expression and activity of insulin-degrading enzyme

Mirian A. Kurauti[1*], José M. Costa-Júnior[1], Sandra M. Ferreira[1], Gustavo J. Santos[1,2], Carlos H. G. Sponton[1], Everardo M. Carneiro[1], Guilherme D. Telles[3], Mara P. T. Chacon-Mikahil[3], Cláudia R. Cavaglieri[3], Luiz F. Rezende[1,4], Antonio C. Boschero[1]

[1]Obesity and Comorbidities Research Center (OCRC), Institute of Biology, University of Campinas (UNICAMP), Campinas, SP, Brazil

[2]Department of Physiological Sciences, Center of Biological Sciences, Federal University of Santa Catarina (UFSC), Florianopolis, SC, Brazil

[3]Exercise Physiology Laboratory (FISEX), Faculty of Physical Education, University of Campinas (UNICAMP), Campinas, SP, Brazil

[4]Laboratory of Health Sciences, Department of Physiopathology, State University of Montes Claros (UNIMONTES), Montes Claros, MG, Brazil

*****Corresponding author: Obesity and Comorbidities Research Center (OCRC), Institute of Biology, University of Campinas (UNICAMP). Rua Carl Von Linnaeus, Bloco Z, zip code 13083864, Campinas, SP, Brazil. Phone number: +55 19 35210015. E-mail: mirian.kurauti@hotmail.com

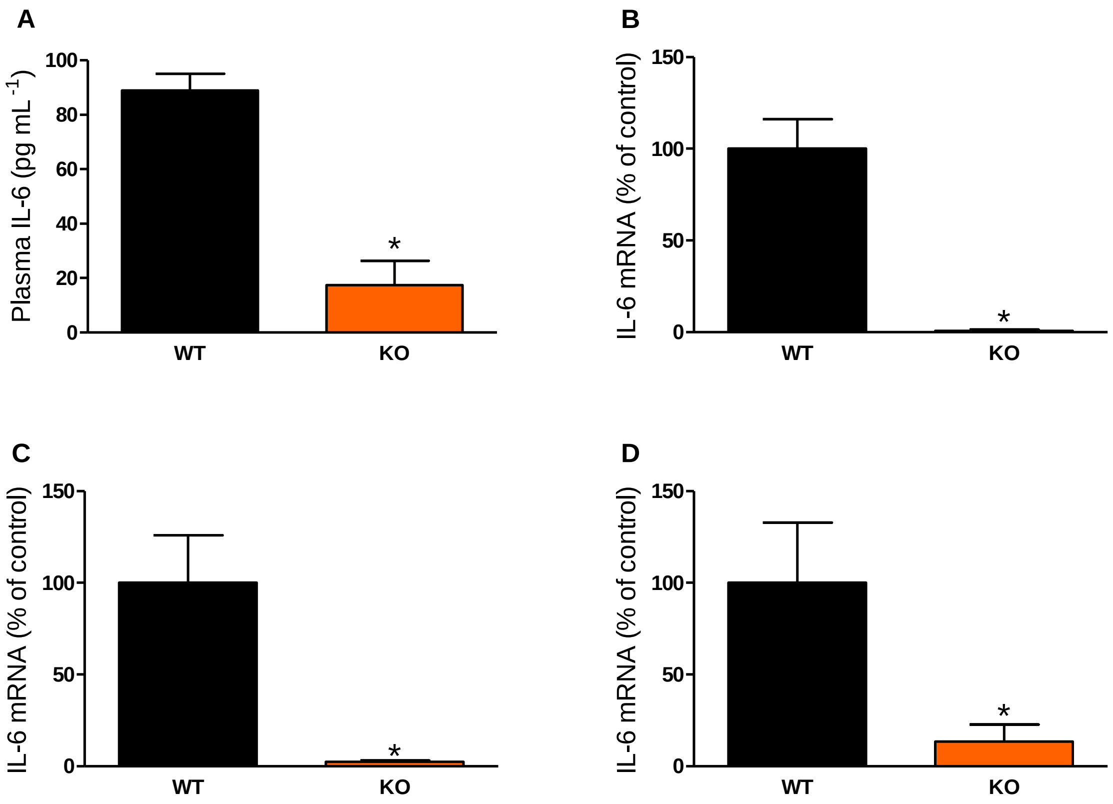

Supplementary figure 1. Plasma concentration and mRNA expression of IL-6. Plasma IL-6 concentration (A) and IL-6 mRNA expression in the liver (B), gastrocnamius skeletal muscle (C) and perigonadal adipose tissue (D). WT, wild type mice; and KO, IL-6 knockout mice. Data are presented as the mean ± S.E.M. n=4. * $p \leq 0.05$ *vs* WT.

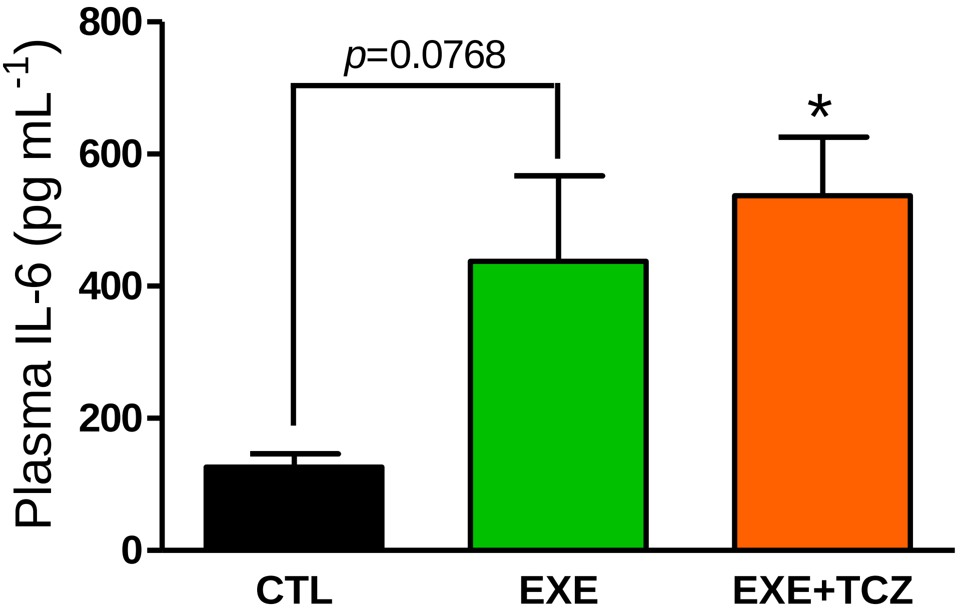

Supplementary figure 2. Plasma IL-6 concentrations in mice after acute exercise. IL-6 concentration in the plasma of mice. CTL, control mice; EXE, exercised mice; and EXE+TCZ, exercised mice treated with 2 mg kg^{-1} Tocilizumab 1-h before the acute exercise protocol (a single bout of exercise on a treadmill inclined at 25° for 3-h at 60-70% of VO_2 max). Data are presented as the mean ± S.E.M. n=3. * $p \leq 0.05$ *vs* CTL.

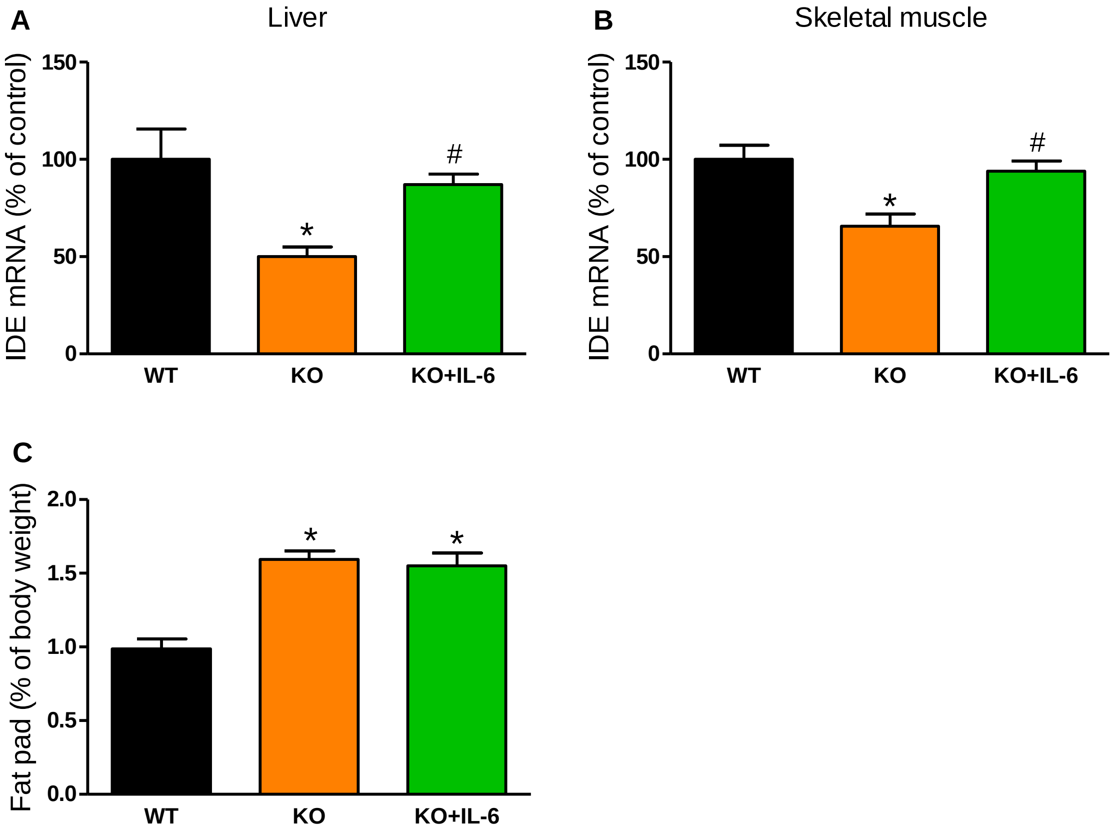

Supplementary figure 3. IDE mRNA expression and fat pad of KO mice after IL-6 treatment. IDE mRNA expression in the liver (A) and gastrocnemius skeletal muscle (B) and percent of body weight of perigonadal fat pad (C) of mice. WT, wild type; KO, IL-6 knockout and KO+IL-6, IL-6 KO mice treated with 200 ng per day IL-6 during 3 days before the experiments. Data are presented as the mean ± S.E.M. n=5-6. * $p < 0.05$ *vs* WT, # $p < 0.05$ *vs* KO.
